# The impact of quetiapine monotherapy or in combination with lithium on the thyroid function in patients with bipolar depression: A retrospective study

**DOI:** 10.1111/cns.14342

**Published:** 2023-07-09

**Authors:** Lingzhuo Kong, Yuting Shen, Shaohua Hu, Jianbo Lai

**Affiliations:** ^1^ Department of Psychiatry, The First Affiliated Hospital Zhejiang University School of Medicine Hangzhou China; ^2^ School of Psychiatry Wenzhou Medical University Wenzhou China; ^3^ The Key Laboratory of Mental Disorder's Management in Zhejiang Province Hangzhou China; ^4^ Brain Research Institute of Zhejiang University Hangzhou China; ^5^ Zhejiang Engineering Center for Mathematical Mental Health Hangzhou China; ^6^ Department of Neurobiology, NHC and CAMS Key Laboratory of Medical Neurobiology, School of Brain Science and Brian Medicine, and MOE Frontier Science Center for Brain Science and Brain‐Machine Integration Zhejiang University School of Medicine Hangzhou China

**Keywords:** autoimmune thyroiditis, bipolar disorder, lithium, quetiapine, thyroid function

## Abstract

**Objective:**

This study aims to investigate whether quetiapine monotherapy or in combination with lithium significantly disturbs thyroid function in depressed patients with bipolar disorder (BD), and whether difference exists in the post‐treatment thyroid function between the two therapies.

**Methods:**

Based on the electric medical records, outpatients and inpatients with a current depressive episode of BD from January 2016 to December 2022 were screened. All patients were treated with quetiapine monotherapy or in combination with lithium. In addition to the demographic data and depression scale, thyroid profiles including total thyroxine (TT4), total triiodothyronine (TT3), free thyroxine (FT4), free triiodothyronine (FT3), thyroid‐stimulating hormone (TSH), thyroid peroxidase antibody (TPOAb), and antithyroglobulin antibody (TGAb) were recorded, analyzed, and compared before and after the treatment.

**Results:**

Totally, 73 eligible patients were enrolled, including 53 in the monotherapy group (MG) and 20 in the combined therapy group (CG). No significant differences in thyroid profiles were detected between the two groups at the baseline (*p* > 0.05). After one‐month treatment, in the MG, serum levels of TT4, TT3, FT4, and FT3 reduced significantly (*p* < 0.05), while TSH, TPOAb, and TGAb increased significantly (*p* < 0.05). In the CG, serum levels of TT4, TT3, and FT4 reduced and TSH increased following one‐month treatment (*p* < 0.05), with no significant change in FT3, TPOAb, or TGAb (*p* > 0.05). After one‐month treatment, no difference of TT4, TT3, FT4, FT3, and TSH was found between the two groups (*p* > 0.05).

**Conclusion:**

Both quetiapine monotherapy and a combined therapy with lithium significantly disturbed thyroid function in patients with bipolar depression, while quetiapine monotherapy seems to be associated with immune dysregulation in the thyroid.

## Graphical abstract

Quetiapine monotherapy or in combination with lithium significantly disrupted the thyroid function in patients with bipolar depression. Lithium supplement appeared to rescue quetiapine‐related immune dysregulation in the thyroid.

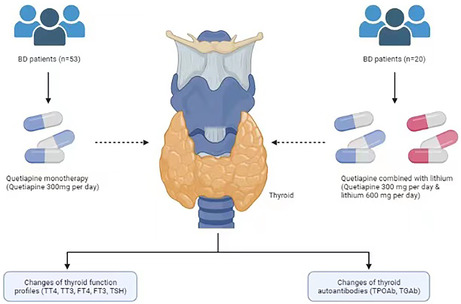

## 1 INTRODUCTION

Bipolar disorder (BD), a disabling psychiatric disorder characterized by recurrence of manic and depressive episodes, affects approximately 1%–4% of the population worldwide,^1^ leading to adverse outcomes including cognitive impairment and suicide, and causing a great burden for the patients and public health institutions.^2^, ^3^

Alternations in thyroid function are possibly bidirectionally associated with emotion fluctuation,^4^ since patients with mood disorders showed a higher comorbidity rate with hyperthyroidism and hypothyroidism, and thyroid dysfunction could result in mood abnormalities.^5^, ^6^, ^7^ Interventions on the hypothalamic–pituitary–thyroid axis might reverse depression.^8^ In particular, it has been reported that compared to the general population, thyroid dysfunction appeared more frequently in BD patients,^5^ which might be implicated in the deficits of BD neuropathology and dysfunction of the hypothalamic–pituitary–thyroid axis.^9^ In addition, research has shown that the prevalence of autoimmune thyroid disease and levels of thyroid autoantibodies in BD patients were significantly higher,^5^, ^10^ indicating a potential linkage between thyroid alternations and BD pathophysiology.

Psychotropic drugs can disturb the process of the synthesis, secretion, transport, metabolism, and absorption of thyroid hormones,^11^ resulting in functional and pathological abnormalities of the thyroid. For example, quetiapine induced hypothyroidism has been widely documented in BD patients,^12^, ^13^ which might probably be related to the competitive metabolism of thyroid hormones and quetiapine via UDP‐glucuronosyltransferase.^14^ Lithium is a classic mood stabilizer for BD and is associated with drug‐induced goiter, hypothyroidism, or rarely hyperthyroidism in some BD cases,^15^, ^16^ by changing the structure of thyroglobulin and inhibiting thyroid hormone release from the thyroid gland.^16^, ^17^ In the clinical practice, the treatment strategy of quetiapine monotherapy or in combination with lithium is widely used among patients with BD. Some studies indicated that a combination of quetiapine and lithium was more effective than either agent alone in emotional maintenance and depressive prevention.^18^ However, to date, no published study has ever reported the thyroid disturbance due to this combined therapy in bipolar patients, neither was it compared with quetiapine monotherapy.

To address these issues, this study aimed to investigate whether a combined therapy of quetiapine and lithium disrupted the thyroid function, and whether differences on disturbing the thyroid function existed between the monotherapy and the combined therapy.

## 2 METHODS

This study was approved by the Clinical Research Ethics Committee of the First Affiliated Hospital, Zhejiang University School of Medicine. Due to the retrospective, noninterventional, and anonymous nature of this study, written informed consent from patients was waived.

Based on the hospital electronic medical system, naturalistic medical information of outpatients and inpatients in the First Affiliated Hospital, Zhejiang University School of Medicine, was extracted from January 2016 to December 2022. Inclusion criteria: (1) 18 ~ 55 years old; (2) drug‐naïve or medication‐free for at least 3 months before the visit to psychiatric department; (3) a discharge diagnosis with BD according to the *Diagnostic and Statistical Manual of Mental Disorders, Fifth Edition* (*DSM‐5*), within a depressive episode; (4) patients receiving quetiapine monotherapy and a combined therapy of quetiapine and lithium were followed up for at least 1 month; (5) no additional psychotropic medication was applied during the one‐month observation for BD monotherapy or combined therapy. Exclusion criteria: (1) comorbidity with other psychiatric disorders, (2) severe physical illnesses that need medications or had an influence on thyroid function, (3) any known autoimmune diseases, and (4) intolerable to quetiapine or lithium.

In the MG, patients received 300 mg quetiapine per day (oral administration at night) initially without other drug intervention. In the CG, patients received 300 mg quetiapine (oral administration at night) and 600 mg lithium (oral administration, 300 mg in the morning and 300 mg at night) initially per day. Closely monitor plasma drug concentrations to avoid exceeding or falling below the therapeutic doses. For all patients enrolled, demographic data (gender, age, height, weight, and disease course) and laboratory tests of thyroid functions (TT4, FT4, TT3, FT3, and TSH) as well as thyroid peroxidase antibody (TPOAb) and antithyroglobulin antibody (TGAb) were collected. Body mass index (BMI) was defined as the weight divided by the square of the height (kg/m^2^). The depression severity before and after the treatment in all patients was assessed by the 24‐item Hamilton Depression Rating Scale (HAMD‐24).

All statistical analyses were performed with the Statistical Package for the Social Sciences software v. 24.0 (SPSS Inc). Patients were divided into two groups: the monotherapy group (MG) and the combined therapy group (CG). Thyroid profiles between baseline and posttreatment were collected and compared respectively, and posttreatment thyroid profiles in the two groups were compared. Shapiro–Wilk test was used to test normality. The categorical data were analyzed by Pearson Chi‐square test. Continuous variable data were analyzed by independent‐ and paired‐samples *t* test (normally distributed data) or rank‐sum test (not normally distributed data). Normally distributed data are presented as mean ± standard deviation (SD); *p*‐value <0.05 was denoted as statistical significance.

## 3 RESULTS

### 3.1 Demographic data

Totally, 73 BD patients presenting with a current depressive episode were eligible in this study (53 patients in the MG and 20 in the CG). The demographic comorbidities including iron‐deficiency anemia, chronic gastritis, eczema, anaphylactic rhinitis, saprodontia, and myopia. None of these comorbidities was known to have a significant impact on thyroid function. The current depressive episode was longer in the CG (*p* < 0.05). No difference was found in the gender, age, weight, height, or total disease course between the two groups. (Table 1).

**TABLE 1:** Demographic and clinical characteristics of the subjects (mean ± SD).

	MG (*n* = 53)	CG (*n* = 20)	*p*‐Value*
Gender (*n*, male/female)	13/40	6/14	0.666
Age (years)	18.02 ± 5.44	22.45 ± 10.15	0.084
Height (centimeter)	164.49 ± 7.27	162.00 ± 8.47	0.217
Weight (kilogram)	54.80 ± 8.60	56.90 ± 10.81	0.389
BMI (kg/m^2^)	20.25 ± 2.91	21.65 ± 3.39	0.122
Total disease course (month)	32.42 ± 27.76	44.40 ± 49.79	0.592
Duration of current depressive episode (day)	93.58 ± 165.95	336.60 ± 423.52	<0.001

*Note*: *Significance at *p* < 0.05 (two‐tailed). Normally distributed data: height (both), weight (both), BMI (MG); not normally distributed data: age (both), BMI (CG), total disease course (both), duration of current depressive episode (both).

Abbreviations: BMI, body mass index.; CG, combined therapy group; MG, monotherapy group.

### 3.2 Baseline thyroid profiles in the two groups

The thyroid profiles of all patients were within the normal limits. No difference of baseline thyroid profiles, including TT4, FT4, TT3, FT3, TSH, TPOAb, or TGAb, was identified between the two groups. (Table 2).

**TABLE 2:** Compassion of baseline thyroid profiles in the two groups (mean ± SD).

	MG (*n* = 53)	CG (*n* = 20)	*p*‐Value*	Normal reference range
TT4 (nmol/L)	95.82 ± 16.43	93.33 ± 15.75	0.561	62.68 ~ 150.85
TT3 (nmol/L)	1.49 ± 0.18	1.45 ± 0.16	0.350	0.98 ~ 2.33
TSH (mIU/L)	1.58 ± 0.95	1.34 ± 0.72	0.407	0.35 ~ 4.94
FT4 (pmol/L)	13.11 ± 1.47	12.88 ± 1.59	0.500	9.01 ~ 19.05
FT3 (pmol/L)	4.45 ± 0.52	4.23 ± 0.38	0.062	2.43 ~ 6.01
TPOAb	0.33 ± 0.24	0.45 ± 0.29	0.061	0.00 ~ 5.61
TGAb	1.03 ± 0.75	1.28 ± 1.57	0.621	0.00 ~ 4.11

*Note*: *Significance at *p* < 0.05 (two‐tailed). Normally distributed data: TT4 (both), TT3 (both), TSH (CG), FT4 (CG), FT3 (both), TPOAb (both), TGAb (MG); not normally distributed data: TSH (MG), FT4 (MG), TGAb (CG).

Abbreviations: CG, combined therapy group; FT3, free triiodothyronine; FT4, free thyroxine; MG, monotherapy group; TSH, thyroid‐stimulating hormone; TT3, total triiodothyronine; TT4, total thyroxine.

### 3.3 Impact of quetiapine monotherapy and quetiapine in combination with lithium on thyroid profiles and thyroid antibodies in depressed BD patients

In the MG, the maintenance dose of quetiapine in five patients was 500 mg per day, another seven was 400 mg per day, and the remaining (*n* = 41) was all 300 mg per day. In the CG, the maintenance dose of quetiapine in two patients was 500 mg per day, another one was 400 mg per day, and the remaining (*n* = 17) was all 300 mg per day. The maintenance dose of lithium carbonate extended‐release tablet in twelve patient was 900 mg per day, and the remaining (*n* = 8) were 600 mg per day. After one‐month treatment, the scores of HAMD‐24 were significantly decreased (*p* < 0.001) in both groups. (Table 3).

**TABLE 3:** Changes in the scores of HAMD‐24 before and after the one‐month treatment (mean ± SD).

	Baseline	Post‐treatment	*p*‐Value*
MG (*n* = 53)	28.83 ± 5.31	13.19 ± 7.42	<0.001
CG (*n* = 20)	27.75 ± 6.06	12.65 ± 7.44	<0.001

*Note*: *Significance at *p* < 0.05 (two‐tailed). Normally distributed data: MG baseline, CG baseline, CG post treatment; not normally distributed data: MG post‐treatment.

Abbreviation: HAMD‐24, 24‐item Hamilton Depression Rating Scale.

**FIGURE 1:**
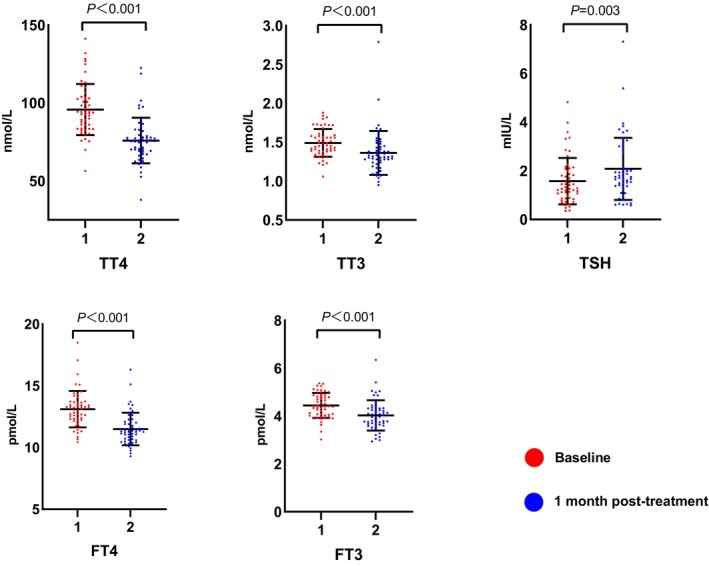
Changes of thyroid profiles before and after one‐month quetiapine monotherapy in depressed patients with BD (*n* = 53). After one‐month quetiapine monotherapy, the serum levels of TT4, TT3, FT4, and FT3 decreased significantly (*p* < 0.05), while levels of TSH increased significantly (*p* < 0.05). Normally distributed data: TT4 baseline, TT3 baseline, FT3 baseline; not normally distributed data: TT4 post‐treatment, TT3 post‐treatment, TSH (both), FT4 (both), FT3 posttreatment. TT4, total thyroxine; TT3, total triiodothyronine; FT4, free thyroxine; FT3, free triiodothyronine; TSH, thyroid‐stimulating hormone.

In the MG, serum values of TT4, TT3, FT4, and FT3 were significantly reduced after one‐month of quetiapine treatment (*p* < 0.05), TSH was increased (*p* = 0.003). (Figure 1) According to the normal reference range, no patient met the diagnostic criteria for hypothyroidism.

**FIGURE 2:**
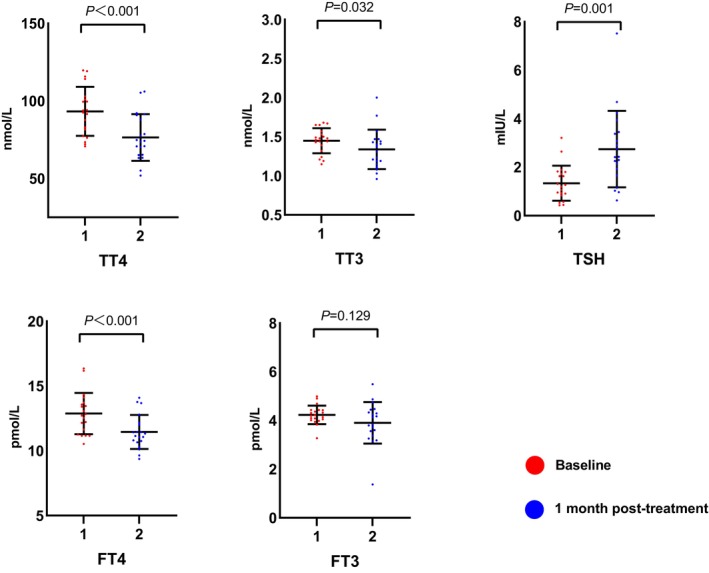
Changes of thyroid profiles before and after one‐month combined therapy of quetiapine and lithium in depressed patients with BD (*n* = 20). After one‐month combined therapy, serum levels of TT4, TT3, and FT4 decreased significantly (*p* < 0.05), while levels of TSH increased significantly (*p* < 0.05). Levels of FT3 showed no significant changes (*p* = 0.129). Normally distributed data: TT4 (both), TT3 (both), TSH baseline, FT4 (both), FT3 (both); not normally distributed data: TSH posttreatment. TT4, total thyroxine; TT3, total triiodothyronine; FT4, free thyroxine; FT3, free triiodothyronine; TSH, thyroid‐stimulating hormone.

In the CG, serum values of TT4, TT3, and FT4 were significantly reduced after one‐month of quetiapine and lithium treatment (*p* < 0.05), TSH was also increased (*p* = 0.001), while FT3 was not significantly changed (*p* = 0.129). (Figure 2) Compared with the normal reference range, no patient was diagnosed with hypothyroidism.

**FIGURE 3:**
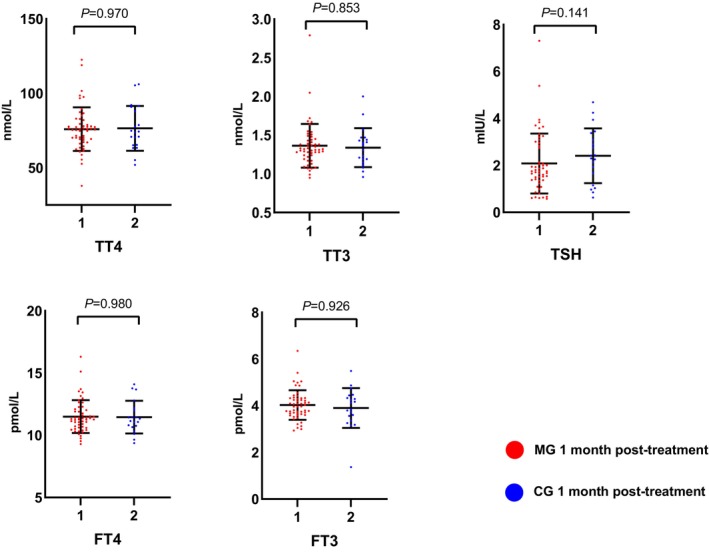
Comparison of the post‐treatment thyroid profiles changes between the two groups. After one‐month of treatment, no significance was noted between the thyroid profiles of the MG and CG (*p* > 0.05). Of note, serum levels of TSH tended to increase in the CG (*p* = 0.141). Normally distributed data: TT4 (CG), TT3 (both), TSH (CG), FT4 (CG), FT3 (both); not normally distributed data: TT4 (MG), TSH (MG), FT4 (MG). CG, combined therapy group; FT4, free thyroxine; FT3, free triiodothyronine; MG, monotherapy group; TT4, total thyroxine; TT3, total triiodothyronine; TSH, thyroid‐stimulating hormone.

**FIGURE 4:**
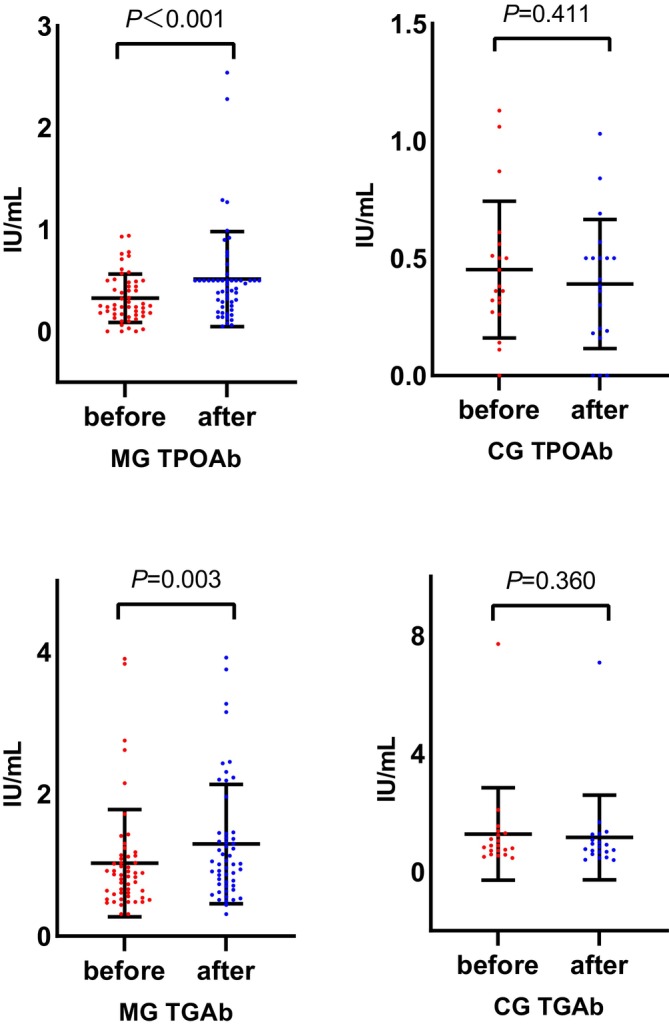
Levels of thyroid peroxidase antibody and antithyroglobulin antibody in the two groups before and after treatment. After one‐month treatment, serum levels of both TPOAb and TGAb increased significantly in the MG (*p* < 0.05). Serum levels of TPOAb and TGAb showed no significance between before and after combined therapy (*p* > 0.05). Importantly, levels of TPOAb and TGAb tended to decrease in the CG, suggesting the potential thyroid‐protective effect of lithium. Normally distributed data: TPOAb (CG, both baseline and posttreatment); not normally distributed data: TPOAb (MG, both baseline and posttreatment), TGAb (MG, both baseline and posttreatment), TGAb (CG, both baseline and posttreatment). CG: combined therapy group; MG: monotherapy group; TPOAb: thyroid peroxidase antibody; TGAb: antithyroglobulin antibody.

In addition, no significant change of thyroid functions was found between the MG and CG after one‐month treatment. (Figure 3) However, serum levels of TPOAb and TGAb increased significantly after quetiapine monotherapy (*p* < 0.05), while no significant change was observed in TPOAb (*p* = 0.411) and TGAb (*p* = 0.360) levels before and after combined treatment. (Figure 4).

## 4 DISCUSSION

In this study, we retrospectively explored the changes in the thyroid profiles following quetiapine monotherapy or combined therapy of quetiapine and lithium in BD patients presenting with a depressive episode. We found that following one‐month of quetiapine monotherapy or combined therapy could increase the serum levels of TSH while reduce other indicators including TT4, TT3, and FT4. In general, these findings suggested that thyroid profiles could be remarkably influenced by short‐term quetiapine monotherapy as well as a combined therapy of quetiapine and lithium. Lithium supplement did not aggravate the adverse effect of quetiapine on thyroid function, and to some extent, alleviate quetiapine‐related elevation in TPOAb and TGAb levels, both of which are known as indicators of autoimmune thyroiditis. Although clinically significant manifestations of thyroid functional changes have not been observed, findings of this study still warrant more attention.

Previous studies have demonstrated the drug‐induced hypothyroidism in monotherapy of quetiapine, or lithium.^12^, ^13^, ^19^, ^20^ Especially, a study reported that after 6 weeks of quetiapine intervention, serum levels of TT4 and TT3 decreased significantly, while levels of TSH increased,^21^ which was consistent with our findings. However, on the one hand, changes in these laboratory indicators are less likely to result in clinically significant symptoms. Indeed, no subjects in our study showed manifestations of hypothyroidism that required further clinical intervention. On the other hand, compared with potential and opportunistic hypothyroidism related to pharmacotherapy, the pharmacological effects of quetiapine and lithium on stabilizing the mood in BD are of more concern. Therefore, despite of the thyroid side effects, these drugs are still the first‐line recommendations for the clinical practice in BD treatment.

Quetiapine is an atypical antipsychotic drug that can antagonize the activity of dopamine D2 receptor and serotonin 5‐HT2A receptor,^22^ while lithium is a commonly used mood stabilizer that inhibits the abnormal release of norepinephrine and dopamine^23^ by maintaining the homeostasis of endoplasmic reticulum in the cerebral cortex and striatum.^24^ Quetiapine can compete with thyroid hormones to bind UDP‐glucuronosyltransferase transferase, resulting in decreased thyroid hormone sensitivity.^14^ Lithium could inhibit the release of thyroid hormone via alteration in tubulin polymerization as well as inhibition of the action of TSH on cyclic adenosine monophosphate,^17^ thus resulting in thyroid function changes. In our study, both MG and CG led to significant changes in thyroid profiles, but no difference was found between the two groups, indicating that at least in the first one‐month pharmacological intervention, lithium would not cause further deterioration of thyroid function on the basis of quetiapine, thus confirming its clinical safety.

In addition, lithium is concentrated three to four times in the thyroid than the peripheral blood, which could be coupled to the iodide concentrating process.^17^, ^25^ Except for the inhibition of norepinephrine and dopamine release, lithium is also thought to stabilize mood by regulating thyroid hormone metabolism in the central nervous system.^26^, ^27^, ^28^ Unfortunately, we did not measure the thyroid iodine uptake rate before and after treatment. In future studies, the thyroid iodine uptake rate of patients before and after treatment is warranted to better clarify the mood‐stabilizing mechanism of lithium in the brain.

Moreover, although existing evidence suggests that thyroid disturbance in BD patients is closely linked to psychiatric disorders, thyroid diseases in turn can also lead to emotional fluctuation and cognition impairment,^29^, ^30^, ^31^ which is labeled as the diagnosis of “bipolar and related disorder due to another medical condition” according to *DSM‐5*.^32^ A systematic review concluded that thyroid autoimmunity was suggested to be an independent risk factor for BD and was even considered as an “endophenotype” of BD.^5^ In addition, low levels of serum FT4 were associated with more frequent mood episodes and severer depression in lithium‐maintained individuals with BD.^33^ Another epidemiological evidence is that BD is closely correlated with autoimmune diseases including autoimmune thyroiditis,^34^, ^35^, ^36^ indicating that systematic autoimmune dysregulation might be a crucial factor for the etiopathogenesis of BD. However, the exact pathophysiological mechanism and the casual relationship still needs further exploration.

Quetiapine could cause autoimmune thyroiditis, as several studies reported elevated autoantibodies including TPOAb and TGAb following quetiapine treatment,^37^, ^38^, ^39^ while discontinuing quetiapine for 2 weeks, levels of these autoantibodies reduced significantly.^37^ In our study, up‐regulated levels of TPOAb and TGAb in the MG were observed, which were consistent with the previous findings. In previous studies, no association has been found between therapeutic dose of lithium exposure and shifting levels of thyroid antibodies in BD patients.^40^, ^41^ In our study, combined quetiapine and lithium appeared to rescue the increasing of serum TPOAb and TGAb due to quetiapine monotherapy, suggesting that lithium might mitigate the immune disturbance in the thyroid induced by quetiapine to some extent.

Notably, “endocrinic thyroid dysfunction” and “autoimmune thyroiditis” are two relatively independent concepts. Autoimmune thyroiditis may not definitely cause thyroid dysfunction, and in turn, thyroid dysfunction is not always associated with thyroid auto‐antibody abnormalities. Although the effect of lithium on the thyroid endocrinic functions has been well recognized, its role in the development of autoimmune thyroiditis remains largely unknown. In this study, we observed that a combined therapy of quetiapine and lithium also disrupted the thyroid functional profiles, but lithium supplement appeared to rescue quetiapine‐related autoimmune thyroiditis. Given that the correlation between lithium exposure and autoimmune thyroiditis is still unclear,^40^, ^41^ more explorations are needed in the future.

### 4.1 Limitations and future directions

Several limitations exist in this study. First, the sample size is relatively small, and retrospective design limited its stringency. Nevertheless, the enrolled patients received no addtional treatment except for the drugs mentioned, and there is also little significant difference in the demographic data, and the study findings herein are still credible. Notably, the duration of current depressive episode in the CG (336.60 ± 423.52 days) was significantly longer than that in the MG (93.58 ± 165.95 days), which might contribute to the difference in thyroid functions between the two groups. A longer depressive duration may cause more significant impairment in social functions, which may explain the supplement of lithium in the CG individuals. It has been reported that lower levels of FT4 and TSH were associated with a longer depressive duration over 3 years.^42^ In our study, we did not find significant difference in baseline thyroid functions between the two groups. Therefore, these results indicated that compared with pharmacological intervention, the duration of depressive episode might not be a predominant influencing factor of thyroid function.

Second, interference factors of thyroid function include sex, disease duration, disease severity, numbers of depressive or manic episodes, underlying diseases, and psychiatric comorbidities. Notably, different states of BD may also influence the thyroid functions. It has been previously reported that the thyroid function varied significantly between manic and depressive episodes of BD.^43^ Serum levels of FT3, TT3, and TT4 in patients with manic episodes (type I BD) changed significantly compared with healthy controls,^44^ and levels of TSH and T4 also varied between bipolar patients with mix and manic states.^45^ In addition, we did not recruit BD patients who received lithium monotherapy in this study, as lithium is more commonly used in controlling manic episode or maintenance phase rather than for treating bipolar depression. These studies suggest that in future research, variables including emotional states and subtypes of BD should be taken into consideration.

Moreover, the one‐month observation period is relatively short, which makes it difficult to monitor the coordinated variation of serum drug concentration and thyroid profiles, and limits the generalization of the study conclusion. In addition, although current evidence does not support that lithium can directly lead to autoimmune thyroiditis,^41^, ^46^, ^47^ lithium has shown a long‐term effect on the thyroid function and even leads to hypothyroidism and clinical or subclinical goiter.^46^, ^48^ Therefore, regular follow‐up (at 6‐ to 12‐month intervals) of thyroid functions during lithium treatment is suggested.^16^ In future studies, longer observation period, more frequent laboratory test, and large simple size are on demand for deeper insight into the influence of pharmacological intervention on thyroid function in BD patients.

Finally, we did not further explore the interaction of lithium and quetiapine. In view of the previous reports that both lithium and quetiapine monotherapy could cause thyroid dysfunction, the combined therapy may lead to further impairment of thyroid function theoretically. However, in our study, lithium supplement not only did not cause deterioration of thyroid function but also tended to reverse the inflammation burden in the thyroid related to quetiapine treatment, indicating the practical safety of the combined therapy. In the future, animal experiment is needed to reproduce this conclusion and further clarify the mechanism.

## 5 CONCLUSION

This retrospective study showed that quetiapine monotherapy and a combined therapy of quetiapine and lithium disrupted the thyroid function in depressed patients with BD, and quetiapine monotherapy seemed to relate to immune dysregulation in the thyroid, which can be reversed by lithium supplement. Taken together, our study provides interesting findings that warrant more attention.

## AUTHOR CONTRIBUTIONS

LZK and YTS carried out data collection, searched literature, did data analysis, prepared figures, and wrote the paper; SHH and JBL conceptualized and edited the paper. All authors read and approved the final manuscript.

## CONFLICT OF INTEREST STATEMENT

No conflicts of interest, financial or otherwise, are declared by the authors.

## Data Availability

The data that support the findings of this study are available from the corresponding author upon reasonable request.
